# Crystal structure and Hirshfeld surface analysis of 2,6-bis­[4-(eth­oxy­carbon­yl)-5-methyl­pyrazol-1-yl]pyridine

**DOI:** 10.1107/S2056989026002860

**Published:** 2026-03-24

**Authors:** Yevhen Krokhmaliuk, Volodymyr M. Fetyukhin, Yuliya M. Davydenko, Oleksandr S. Vynohradov, Vadim O. Pavlenko, Mircea-Odin Apostu

**Affiliations:** a Taras Shevchenko National University of Kyiv, Department of Chemistry, 64 str., Volodymyrska, Kyiv 01601, Ukraine; b I. F. Lab Ltd., Representative of Life Chemicals Inc., Kyiv, Ukraine, 5 Academician Kukhar St., 02094 Kyiv, Ukraine; c Department of Chemistry, Faculty of Chemistry, Al. I. Cuza University of Iasi, 11 Carol I Blvd, Iasi 700506, Romania; University of Buenos Aires, Argentina

**Keywords:** crystal structure, 2,6-bis­(pyrazol-1-yl)pyridine derivatives, pyrazol­yl, Hirshfeld surface analysis, organic synthesis, hydrogen bonding

## Abstract

The title compound (systematic name: ethyl 1-{6-[4-(eth­oxy­carbon­yl)-5-methyl­pyrazol-1-yl]pyridin-2-yl}-5-methyl­pyrazole-4-carboxyl­ate), C_19_H_21_N_5_O_4_, consists of a central pyridine ring substituted by two functionalized pyrazole rings and crystallizes in the centrosymmetric space group *C*2/*c*. The pyridine–pyrazole and pyrazole–pyrazole dihedral angles are 30.55 (5) and 50.81 (8)°, respectively, indicating significant deviations from coplanarity. An intra­molecular C—H⋯O hydrogen bond stabilizes the mol­ecular conformation. In the crystal, mol­ecules form columns along the *c*-axis, but large centroid separations and offsets between parallel pyridine rings contribute to the absence of π–π stacking. Inter­molecular C—H⋯N and C—H⋯O hydrogen bonds link mol­ecules into a three-dimensional network. Hirshfeld surface analysis shows that H⋯H contacts dominate the packing (49.9%), with hydrogen-involving inter­actions contributing over 90% of all contacts. The mol­ecular shape is moderately anisotropic, with globularity and asphericity values of 0.677 and 0.395, respectively. These results highlight the key role of hydrogen-based inter­actions in directing the supra­molecular organization and crystal cohesion.

## Abstract 2

The synthesis and crystal structure of a new organic compound, 2,6-bis­[4-(eth­oxy­carbon­yl)-5-methyl­pyrazol-1-yl]pyridine is reported. A detailed analysis of the inter­molecular contacts in the crystal packing was performed using Hirshfeld surface analysis.

## 1. Chemical context

Complexes based on the ligand 2,6-di(1*H*-pyrazol-1-yl)pyridine attract attention due to their application in coordination chemistry (Halcrow & Kilner, 2003 ▸; Jia, 2011 ▸). In particular, iron complexes exhibit catalytic activity (Magubane *et al.*, 2016 ▸), cross-spin behavior (Pritchard *et al.*, 2009 ▸), and manifestations of the Jahn-Teller effect (Kershaw Cook *et al.*, 2015 ▸). Iron(II) complexes with diethyl 1,1′-(pyridine-2,6-di­yl)*bis*(1*H*-pyrazole-4-carboxyl­ate) demonstrate spin crossover properties that can be induced thermally and by light through the LIESST (light-induced excited spin state trapping) mechanism (García-López *et al.*, 2023 ▸). Moreover, the spin state can be reversibly modulated by guest mol­ecules such as MeNO_2_, MeCN, Me_2_CO, and MeCOOH. Considering the coordination versatility and functional potential of pyrazolyl­pyridine ligands, we aimed to design and synthesize a novel methyl-substituted derivative of 2,6-di(1*H*-pyrazol-1-yl)pyridine and to investigate its structural features using single-crystal X-ray diffraction.
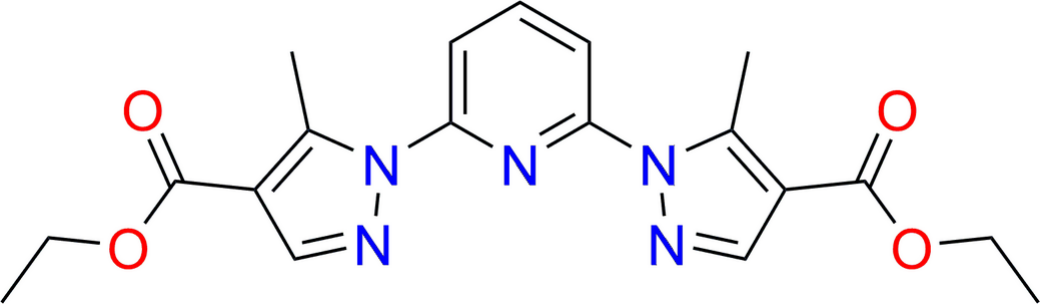


## 2. Structural commentary

**Figure 1:**
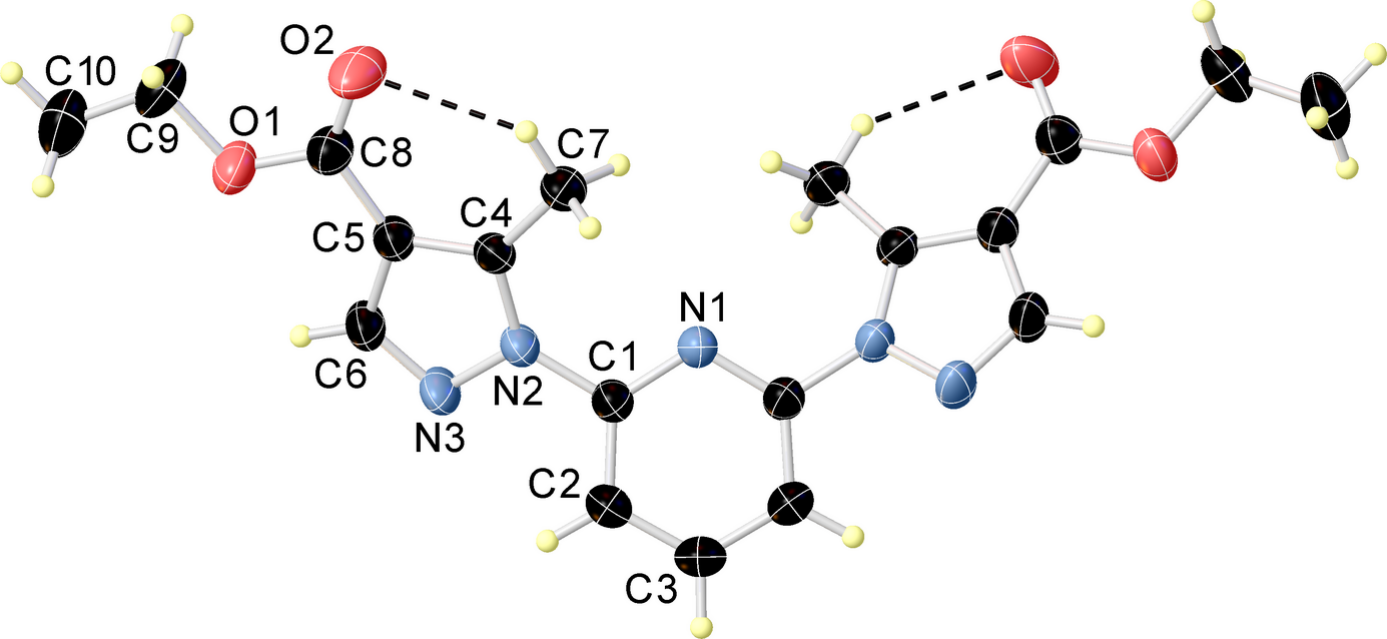
The mol­ecular structure of the title compound, with displacement ellipsoids drawn at the 50% probability level.

In the title compound, C_19_H_21_N_5_O_4_, the polycyclic system is composed of three parts: one central pyridine ring substituted by two functionalized pyrazole rings (Fig. 1 ▸). The mol­ecule is centrosymmetric with a crystallographic twofold rotation axis (*C*_2_) passing through the N1 and C3 atoms of the central pyridine ring. The dihedral angle between the planes of the pyridine ring and the adjacent pyrazole fragment is 30.55 (5)°, indicating a significant deviation from coplanarity between the two aromatic systems. The dihedral angle between the planes containing two pyrazole rings is 50.81 (8)°. Furthermore, the distance between the centroids of the pyridine ring plane (C1–C3, N1) and one of the pyrazole ring planes (C4–C6, N2, N3) is 3.8535 (7) Å, whereas the centroid-to-centroid distance between the two symmetrically positioned pyrazole moieties is 6.6980 (12) Å. The C8=O2 and C8—O1 bond lengths of 1.2024 (12) and 1.3349 (18) Å, respectively, are in the expected ranges (Cambridge Structural Database; Groom *et al.*, 2016 ▸). The mol­ecule is stabilized by an intra­molecular C7—H7*B*⋯O2 hydrogen bond. Selected geometric parameters are given in Table 1 ▸.

## 3. Supra­molecular features

**Figure 2:**
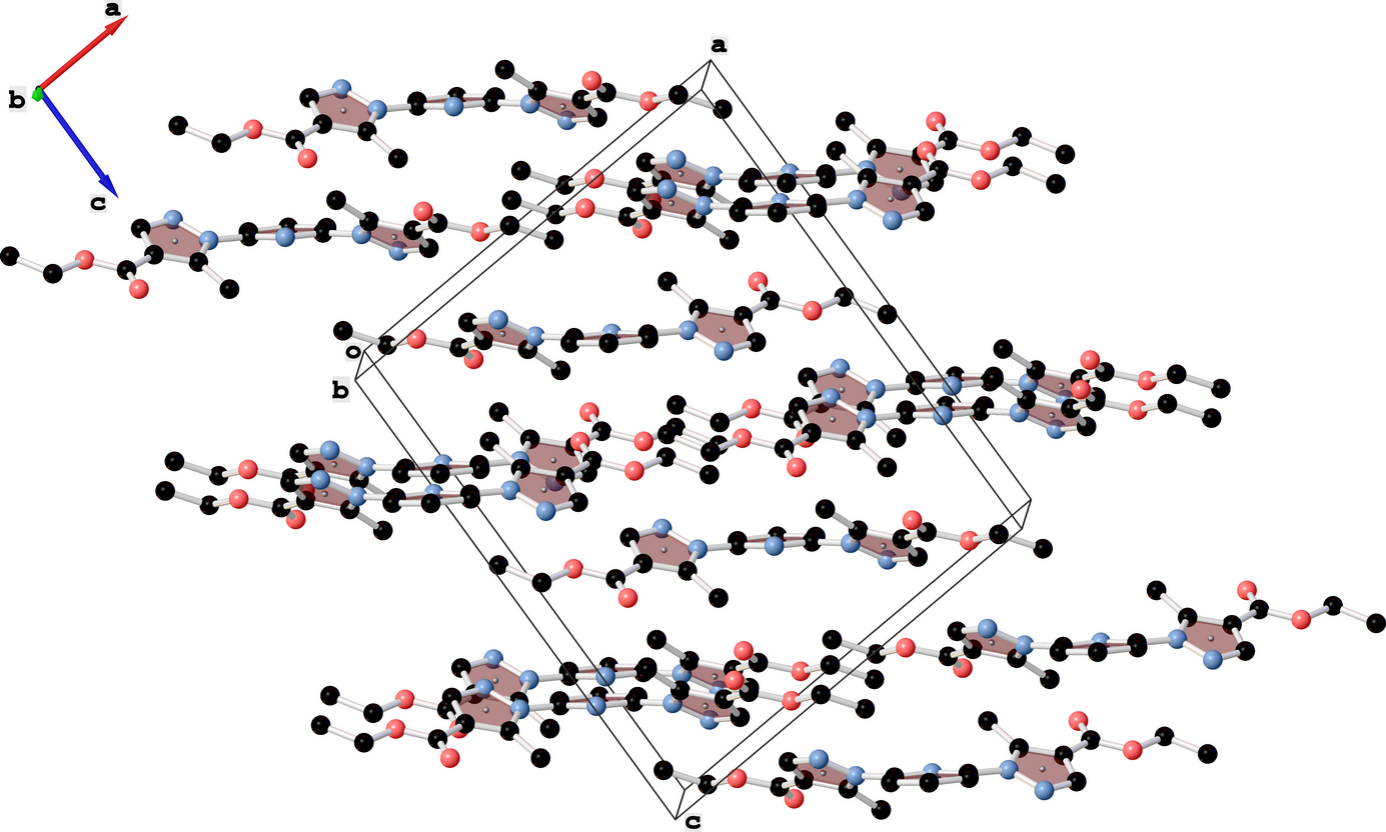
Crystal packing of the title compound viewed along the crystallographic *b* axis. The planes of the pyridine and pyrazole rings are highlighted in red. All hydrogen atoms are omitted for clarity.

In the crystal structure, the mol­ecules are arranged in columns running along the crystallographic *c*-axis (Fig. 2 ▸. Despite the parallel orientation of adjacent pyridine ring planes [twist angle = 0.00 (11)°], the centroid-to-centroid distance between them is 8.0879 (2) Å, and the offset is 6.924 (3) Å, which are significantly larger than the values typically associated with effective π–π stacking inter­actions. These structural parameters clearly indicate the absence of π–π stacking in the crystal. In the crystal, adjacent mol­ecules are linked by C—H⋯N and C—H⋯O hydrogen bonds (Table 2 ▸).

## 4. Database survey

A search of the Cambridge Structural Database using the WebCSD inter­face (CSD version 2025.1, May 2025 release; Groom *et al.*, 2016 ▸) for the C_5_N pyridine ring substituted in the 2- and 6-positions by C_3_N_2_ pyrazole rings, each bearing a –COO fragment at the 4-position, gave 27 hits. The most closely related structure is UGIPIM (Martinez–Martin *et al.*, 2020 ▸), 1,1′-(pyridine-2,6-di­yl)bis­(1*H*-pyrazole-4-carb­oxy­lic acid) aceto­nitrile solvate. Other similar compounds include coordination complexes of iron with this ligand: XORDEQ, and XORDIU (García-López *et al.*, 2019 ▸), as well as a series of iron complexes with diethyl 1,1′-(pyridine-2,6-di­yl)bis­(1*H*-pyrazole-4-carboxyl­ate) [MIHMID, MIHMUP, MIHNAW, MIHNIE, MIHPAY, MIHPEC, MIHPIG (García-López *et al.*, 2023 ▸), TUFXOI (Pritchard *et al.*, 2009 ▸)]. The vast majority of the complexes with the above-mentioned ligands are mononuclear species of the general formula Me*L*_2_.

## 5. Hirshfeld surface analysis

**Figure 3:**
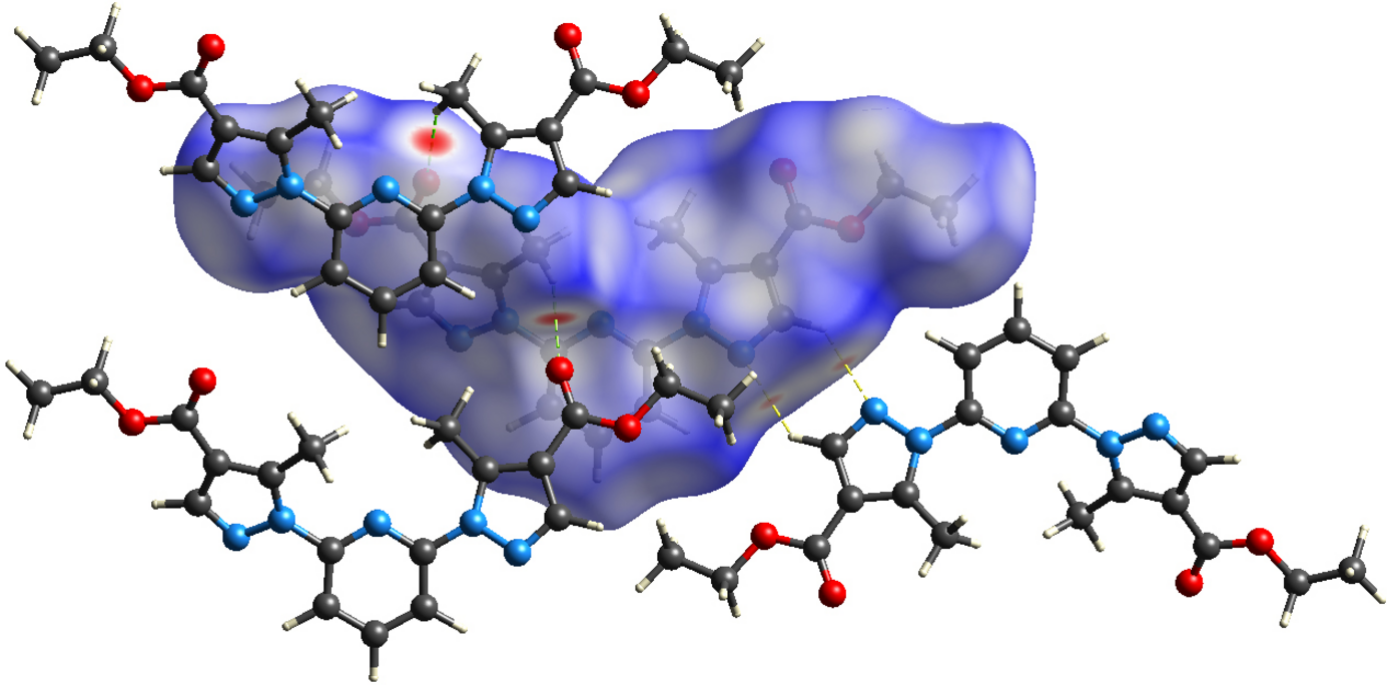
View of the Hirshfeld surface mapped over *d*_norm_ for the title compound showing C—H⋯O and C—H⋯N hydrogen bonds, indicated by green and yellow dashed lines, respectively.

**Figure 5:**
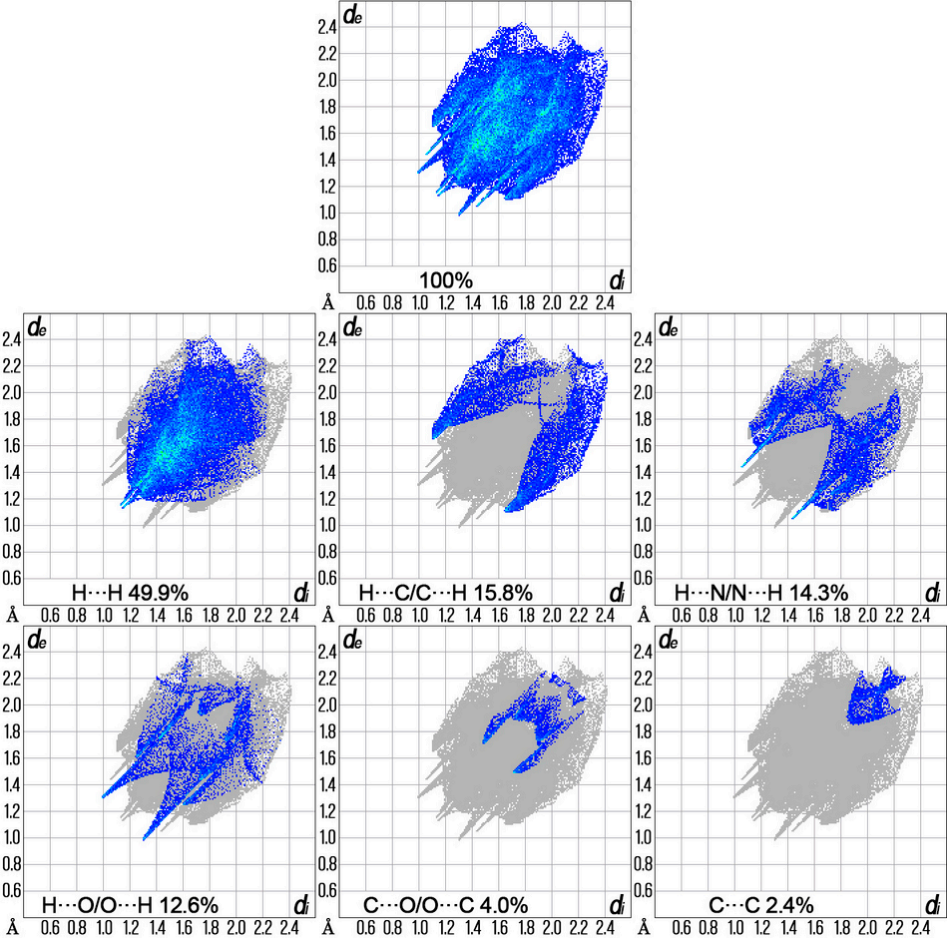
The overall two-dimensional fingerprint plot and those delineated into specified inter­actions.

The Hirshfeld surface analysis and the associated two-dimensional fingerprint plots were performed using *Crystal Explorer 21.5* software (Spackman *et al.*, 2021 ▸), with a standard resolution of the three-dimensional *d*_norm_ surfaces plotted over a fixed colour scale of −0.2207 (red) to 1.2076 (blue) a.u. Eight red spots are observed on the *d*_norm_ surface. The four dark-red regions correspond to short inter­atomic contacts and reflect negative *d*_norm_ values, whereas the remaining four light-red spots indicate weaker inter­molecular inter­actions. The Hirshfeld surfaces mapped over *d*_norm_ are shown for the H⋯H, H⋯C/C⋯H, H⋯N/N⋯H, and H⋯O/O⋯H (Figs. 3 ▸ and 4 ▸), the overall two-dimensional fingerprint plot and the decomposed two-dimensional fingerprint plots are given in Fig. 5 ▸. The shortest inter­molecular contacts are two pairs of C7–H7*A*⋯O2 inter­actions with a length of 2.311 Å, which correspond to contacts between a methyl hydrogen atom of the pyrazole fragment and the carbonyl oxygen atom of the ester (COOEt) group of a neighboring mol­ecule. Considering the weak and predominantly electrostatic character of the inter­molecular C—H⋯O hydrogen bonds, their contribution to the stabilization of the crystal packing is minor. Additionally, two pairs of C6—H6⋯N3 contacts measuring 2.512 Å are observed, representing inter­actions between the hydrogen and nitro­gen atoms of pyrazole fragments from adjacent mol­ecules. H⋯H contacts make 49.9% contribution, which is mostly associated with terminal positions of H atoms and is chemically insignificant. The most significant meaningful inter­actions to the overall crystal packing are from H⋯C/C⋯H (15.8%), H⋯N/N⋯H (14.3%), and H⋯O/O⋯H (12.6%) contacts. There is a small contribution from C⋯O/O⋯C (4.0%) and C⋯C (2.4%), O⋯O (0.4%) and O⋯N/N⋯O (0.5%) weak inter­molecular contacts. The relative percentage contributions to the overall Hirshfeld surface by elements: H⋯all atoms – 68.7%, C⋯all atoms – 13.3%, O⋯all atoms – 9.6% and N⋯all atoms – 8.4%. The data clearly highlight the dominant role of hydrogen-involving inter­actions in the formation and stabilization of the crystal packing. The overwhelming contribution from H⋯H and other hydrogen-related contacts accounts for over 90% of all inter­molecular inter­actions. These findings indicate that hydrogen-based inter­actions are the principal driving force behind the supra­molecular organization and efficient mol­ecular packing within the crystal lattice. The calculated qu­anti­tative physical properties of the Hirshfeld surface — mol­ecular volume (470.13 Å^3^), surface area (431.66 Å^2^), globularity (0.677), and asphericity (0.395) — provide insights into the mol­ecular shape and packing characteristics. The moderate asphericity value indicates a noticeable deviation from spherical symmetry, suggesting that the mol­ecular shape is somewhat elongated or irregular. In addition, the globularity value significantly below 1 implies that the mol­ecular surface is less compact and more complex than a perfect sphere. These parameters suggest an anisotropic and non-spherical mol­ecular shape, which correspondingly influences the packing of mol­ecules in the crystal.

## 6. Synthesis and crystallization

Ethyl 3-oxo­butano­ate (2.06 g, 15.8 mmol) and 1,1-dimeth­oxy-*N*,*N*-di­methyl­methanamine (2.56 g, 17.4 mmol) were placed in a round-bottom flask and refluxed for 2 h. After cooling to room temperature, acetic acid (28 mL) and freshly prepared 2,6-dihydrazinyl­pyridine (Brien *et al.*, 2006 ▸) (1.00 g, 7.2 mmol) were added, and the solution was refluxed overnight. The reaction mixture was evaporated under reduced pressure, and the residue was dissolved in di­chloro­methane. The organic layer was extracted twice with a saturated aqueous solution of NaHCO_3_. The organic phase was dried over Na_2_SO_4_ and evaporated under reduced pressure. The crude product was purified by flash chromatography using a gradient of EtOAc/Hex (10:1 to 1:1, *v*/*v*). As a result, diethyl 1,1′-(pyridine-2,6-di­yl)bis­(5-methyl-1*H*-pyrazole-4-carboxyl­ate) was obtained as a yellow powder (2.4 g, 87%). ^1^H NMR (400 MHz, chloro­form-*d*) δ 8.13–7.99 (*m*, 3H, Ar-H), 7.85 (*d*, *J* = 8.1 Hz, 2H, Ar-H), 4.34 (*q*, *J* = 7.1 Hz, 4H, CH_2_), 2.90 (*s*, 6H, CH_3_), 1.38 (*t*, *J* = 7.1 Hz, 6H, CH_3_); ^13^C NMR (101 MHz, chloro­form-*d*) δ 163.62, 150.97, 145.14, 142.86, 141.31, 116.62, 114.59, 60.29, 14.51, 13.17; m. p. 427 K;

Clear, pale-yellow prismatic crystals suitable for X-ray diffraction were obtained from an Et_2_O/CH_2_Cl_2_ solution by evaporation in the open air.

## 7. Refinement

Crystal data, data collection and structure refinement details are summarized in Table 3 ▸. All hydrogen atoms were positioned geometrically and refined isotropically using a riding model with C—H = 0.96 Å for CH_3_ groups, 0.97 Å for CH_2_ groups, and 0.93 Å for CH groups. The isotropic displacement parameters were set at *U*_iso_(H) = 1.5 *U*_eq_(C) for methyl hydrogens and *U*_iso_(H) = 1.2 *U*_eq_(C) for all other hydrogen atoms.

## Supplementary Material

Crystal structure: contains datablock(s) I. DOI: 10.1107/S2056989026002860/vu2017sup1.cif

Structure factors: contains datablock(s) I. DOI: 10.1107/S2056989026002860/vu2017Isup2.hkl

Scheme of the synthesis of the title compound. DOI: 10.1107/S2056989026002860/vu2017sup3.tif

Supporting information file. DOI: 10.1107/S2056989026002860/vu2017Isup4.cml

CCDC reference: 2539018

Additional supporting information:  crystallographic information; 3D view; checkCIF report

## Figures and Tables

**Figure 4:**
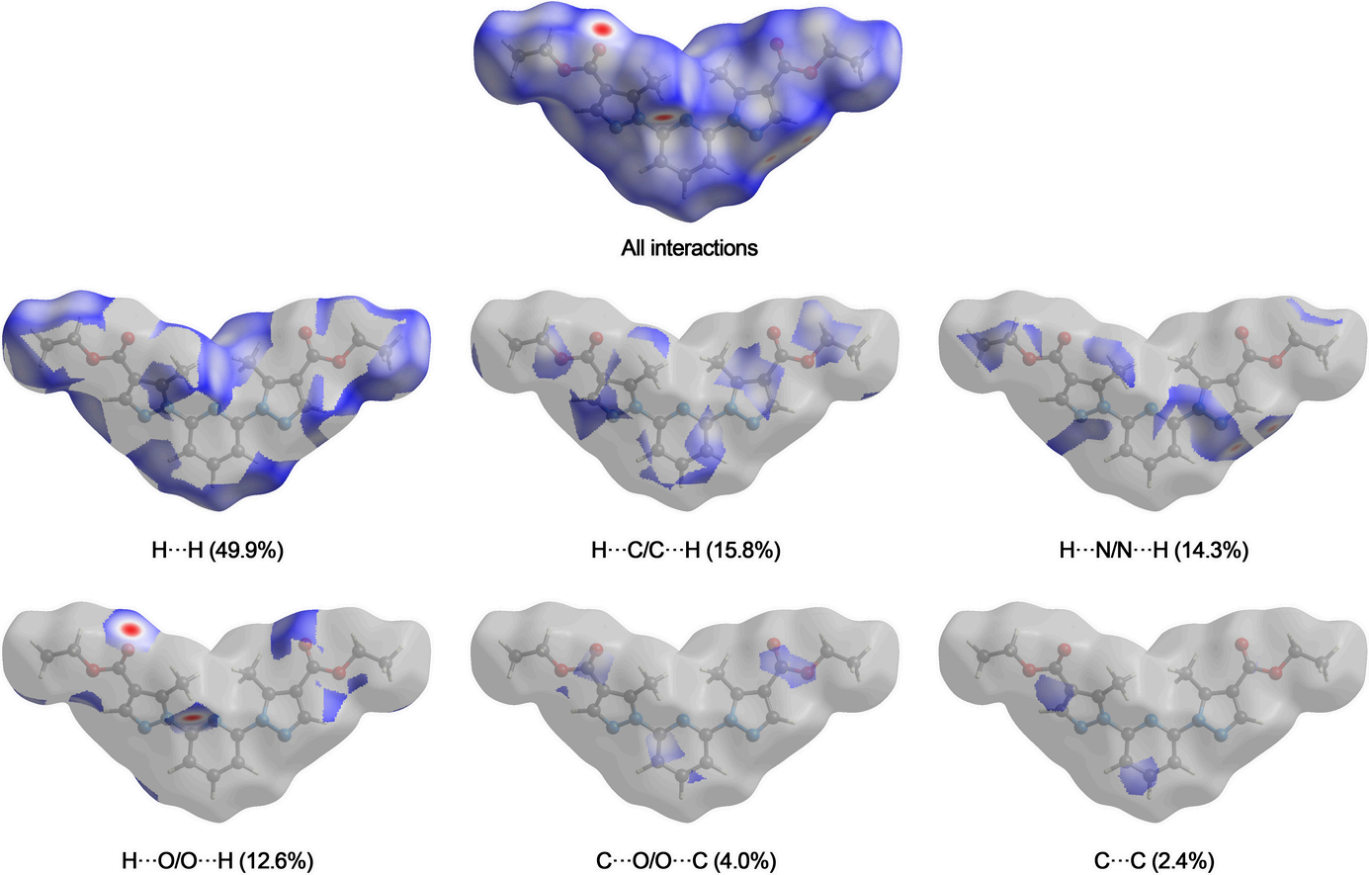
Hirshfeld surface representations with the function *d*_norm_ plotted onto the surface for individual inter­actions.

**Table 1:** Selected geometric parameters (Å, °)

O1—C8	1.3349 (18)	N2—C1	1.4206 (17)
N1—C1	1.3253 (15)	O2—C8	1.2024 (18)
N2—N3	1.3766 (15)		
			
C1—N1—C1^i^	116.65 (16)	C2—C1—N2	118.89 (12)
N3—N2—C1	116.58 (11)	O2—C8—O1	123.32 (14)
C5—C4—C7	129.91 (13)	O2—C8—C5	126.21 (14)
N1—C1—N2	116.61 (12)		

Symmetry code: (i) 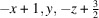.

**Table 2:** Hydrogen-bond geometry (Å, °)

*D*—H⋯*A*	*D*—H	H⋯*A*	*D*⋯*A*	*D*—H⋯*A*
C7—H7*B*⋯O2	0.96	2.46 (1)	3.134 (2)	127 (1)
C7—H7*A*⋯O2	0.96	2.42 (1)	3.290 (2)	151 (1)
C6—H6⋯N3^ii^	0.93	2.63 (1)	3.388 (2)	140 (1)

Symmetry code: (ii) 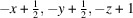.

**Table 3:** Experimental details

Crystal data	
Chemical formula	C_19_H_21_N_5_O_4_
*M*_r_	383.41
Crystal system, space group	Monoclinic, *C*2/*c*
Temperature (K)	293
*a*, *b*, *c* (Å)	13.8701 (5), 8.3233 (3), 16.6408 (6)
β (°)	94.279 (3)
*V* (Å^3^)	1915.76 (12)
*Z*	4
Radiation type	Mo *K*α
μ (mm^−1^)	0.10
Crystal size (mm)	0.3 × 0.2 × 0.2

Data collection	
Diffractometer	Xcalibur, Eos
Absorption correction	Multi-scan (*CrysAlis PRO*; Rigaku OD, 2025 ▸)
*T*_min_, *T*_max_	0.988, 1.000
No. of measured, independent and observed [*I* > 2σ(*I*)] reflections	6654, 2258, 1846
*R*_int_	0.017
(sin θ/λ)_max_ (Å^−1^)	0.689

Refinement	
*R*[*F*^2^ > 2σ(*F*^2^)], *wR*(*F*^2^), *S*	0.047, 0.121, 1.06
No. of reflections	2258
No. of parameters	131
H-atom treatment	H-atom parameters constrained
Δρ_max_, Δρ_min_ (e Å^−3^)	0.21, −0.15

Computer programs: *CrysAlis PRO* (Rigaku OD, 2025 ▸), *SHELXT* (Sheldrick, 2015*a* ▸), *SHELXL2019/3* (Sheldrick, 2015*b* ▸) and *OLEX2* (Dolomanov *et al.*, 2009 ▸).

## supplementary crystallographic information

### Ethyl 1-{6-[4-(ethoxycarbonyl)-5-methylpyrazol-1-yl]\ pyridin-2-yl}-5-methylpyrazole-4-carboxylate Crystal data

**Table:**

C_19_H_21_N_5_O_4_	*F*(000) = 808
*M**_r_* = 383.41	*D*_x_ = 1.329 Mg m^−^^3^
Monoclinic, *C*2/*c*	Mo *K*α radiation, λ = 0.71073 Å
*a* = 13.8701 (5) Å	Cell parameters from 3164 reflections
*b* = 8.3233 (3) Å	θ = 2.5–28.2°
*c* = 16.6408 (6) Å	µ = 0.10 mm^−^^1^
β = 94.279 (3)°	*T* = 293 K
*V* = 1915.76 (12) Å^3^	Prism, clear light colourless
*Z* = 4	0.3 × 0.2 × 0.2 mm

### Ethyl 1-{6-[4-(ethoxycarbonyl)-5-methylpyrazol-1-yl]\ pyridin-2-yl}-5-methylpyrazole-4-carboxylate Data collection

**Table:**

Xcalibur, Eos diffractometer	2258 independent reflections
Radiation source: fine-focus sealed X-ray tube, Enhance (Mo) X-ray Source	1846 reflections with *I* > 2σ(*I*)
Graphite monochromator	*R*_int_ = 0.017
Detector resolution: 16.1593 pixels mm^-1^	θ_max_ = 29.3°, θ_min_ = 2.5°
ω scans	*h* = −17→19
Absorption correction: multi-scan (CrysAlisPro; Rigaku OD, 2025)	*k* = −10→9
*T*_min_ = 0.988, *T*_max_ = 1.000	*l* = −21→22
6654 measured reflections	

### Ethyl 1-{6-[4-(ethoxycarbonyl)-5-methylpyrazol-1-yl]\ pyridin-2-yl}-5-methylpyrazole-4-carboxylate Refinement

**Table:**

Refinement on *F*^2^	Hydrogen site location: inferred from neighbouring sites
Least-squares matrix: full	H-atom parameters constrained
*R*[*F*^2^ > 2σ(*F*^2^)] = 0.047	*w* = 1/[σ^2^(*F*_o_^2^) + (0.0553*P*)^2^ + 0.863*P*] where *P* = (*F*_o_^2^ + 2*F*_c_^2^)/3
*wR*(*F*^2^) = 0.121	(Δ/σ)_max_ < 0.001
*S* = 1.06	Δρ_max_ = 0.21 e Å^−^^3^
2258 reflections	Δρ_min_ = −0.15 e Å^−^^3^
131 parameters	Extinction correction: SHELXL-2019/2 (Sheldrick 2015b), Fc^*^=kFc[1+0.001xFc^2^λ^3^/sin(2θ)]^-1/4^
0 restraints	Extinction coefficient: 0.0103 (11)
Primary atom site location: dual	

### Ethyl 1-{6-[4-(ethoxycarbonyl)-5-methylpyrazol-1-yl]\ pyridin-2-yl}-5-methylpyrazole-4-carboxylate Special details

**Table:**

Geometry. All esds (except the esd in the dihedral angle between two l.s. planes) are estimated using the full covariance matrix. The cell esds are taken into account individually in the estimation of esds in distances, angles and torsion angles; correlations between esds in cell parameters are only used when they are defined by crystal symmetry. An approximate (isotropic) treatment of cell esds is used for estimating esds involving l.s. planes.

### Ethyl 1-{6-[4-(ethoxycarbonyl)-5-methylpyrazol-1-yl]\ pyridin-2-yl}-5-methylpyrazole-4-carboxylate Fractional atomic coordinates and isotropic or equivalent isotropic displacement parameters (Å^2^)

**Table:**

	*x*	*y*	*z*	*U*_iso_*/*U*_eq_	
O1	0.12747 (8)	0.67265 (14)	0.53552 (7)	0.0531 (3)	
N1	0.500000	0.37887 (19)	0.750000	0.0334 (4)	
N2	0.35918 (8)	0.38495 (14)	0.66370 (7)	0.0362 (3)	
O2	0.19474 (10)	0.82118 (15)	0.63654 (8)	0.0689 (4)	
N3	0.31854 (9)	0.31425 (15)	0.59446 (8)	0.0455 (3)	
C4	0.32038 (9)	0.53186 (16)	0.67745 (8)	0.0336 (3)	
C5	0.25205 (10)	0.55721 (17)	0.61385 (8)	0.0375 (3)	
C1	0.43158 (9)	0.29526 (17)	0.70887 (8)	0.0356 (3)	
C6	0.25485 (11)	0.41904 (19)	0.56543 (9)	0.0450 (4)	
H6	0.215840	0.403902	0.518117	0.054*	
C7	0.34643 (11)	0.63313 (18)	0.74956 (9)	0.0428 (4)	
H7A	0.339730	0.571246	0.797503	0.064*	
H7B	0.304232	0.724593	0.749239	0.064*	
H7C	0.412121	0.668872	0.748431	0.064*	
C8	0.19070 (11)	0.69797 (18)	0.59872 (9)	0.0422 (4)	
C2	0.42718 (12)	0.12910 (19)	0.70670 (10)	0.0507 (4)	
H2	0.377389	0.075376	0.677365	0.061*	
C3	0.500000	0.0471 (3)	0.750000	0.0602 (7)	
H3	0.500001	−0.064607	0.749999	0.072*	
C9	0.05946 (13)	0.7998 (2)	0.51338 (10)	0.0561 (5)	
H9A	0.093542	0.898624	0.503183	0.067*	
H9B	0.017386	0.818789	0.556416	0.067*	
C10	0.00211 (15)	0.7460 (3)	0.43922 (12)	0.0757 (6)	
H10A	0.044512	0.728822	0.396993	0.113*	
H10B	−0.044625	0.826846	0.422786	0.113*	
H10C	−0.030589	0.647511	0.449980	0.113*	

### Ethyl 1-{6-[4-(ethoxycarbonyl)-5-methylpyrazol-1-yl]\ pyridin-2-yl}-5-methylpyrazole-4-carboxylate Atomic displacement parameters (Å^2^)

**Table:**

	*U*^11^	*U*^22^	*U*^33^	*U*^12^	*U*^13^	*U*^23^
O1	0.0482 (6)	0.0533 (7)	0.0554 (7)	0.0152 (5)	−0.0126 (5)	−0.0053 (5)
N1	0.0312 (8)	0.0322 (8)	0.0365 (8)	0.000	−0.0007 (6)	0.000
N2	0.0338 (6)	0.0347 (6)	0.0389 (6)	0.0012 (5)	−0.0048 (5)	−0.0082 (5)
O2	0.0894 (10)	0.0520 (8)	0.0620 (8)	0.0260 (7)	−0.0153 (7)	−0.0174 (6)
N3	0.0445 (7)	0.0438 (7)	0.0459 (7)	0.0054 (6)	−0.0125 (6)	−0.0168 (5)
C4	0.0322 (7)	0.0317 (7)	0.0369 (7)	−0.0019 (5)	0.0018 (5)	−0.0033 (5)
C5	0.0341 (7)	0.0392 (8)	0.0385 (7)	0.0012 (6)	−0.0011 (5)	−0.0060 (6)
C1	0.0342 (7)	0.0348 (7)	0.0372 (7)	0.0003 (5)	−0.0003 (6)	−0.0032 (5)
C6	0.0399 (8)	0.0477 (9)	0.0453 (8)	0.0045 (6)	−0.0110 (6)	−0.0129 (7)
C7	0.0487 (8)	0.0381 (8)	0.0405 (8)	0.0033 (6)	−0.0042 (6)	−0.0079 (6)
C8	0.0425 (8)	0.0454 (9)	0.0386 (7)	0.0067 (6)	0.0023 (6)	−0.0028 (6)
C2	0.0522 (9)	0.0356 (8)	0.0615 (10)	−0.0038 (7)	−0.0137 (8)	−0.0054 (7)
C3	0.0675 (16)	0.0306 (12)	0.0790 (17)	0.000	−0.0187 (13)	0.000
C9	0.0505 (9)	0.0638 (11)	0.0537 (9)	0.0222 (8)	0.0017 (8)	0.0101 (8)
C10	0.0643 (12)	0.0864 (15)	0.0725 (13)	0.0117 (11)	−0.0204 (10)	0.0125 (11)

### Ethyl 1-{6-[4-(ethoxycarbonyl)-5-methylpyrazol-1-yl]\ pyridin-2-yl}-5-methylpyrazole-4-carboxylate Geometric parameters (Å, º)

**Table:**

O1—C8	1.3349 (18)	C6—H6	0.9300
O1—C9	1.4469 (19)	C7—H7A	0.9600
N1—C1^i^	1.3253 (15)	C7—H7B	0.9600
N1—C1	1.3253 (15)	C7—H7C	0.9600
N2—N3	1.3766 (15)	C2—H2	0.9300
N2—C4	1.3622 (17)	C2—C3	1.3770 (19)
N2—C1	1.4206 (17)	C3—H3	0.9300
O2—C8	1.2024 (18)	C9—H9A	0.9700
N3—C6	1.3080 (19)	C9—H9B	0.9700
C4—C5	1.3832 (18)	C9—C10	1.487 (3)
C4—C7	1.4886 (18)	C10—H10A	0.9600
C5—C6	1.406 (2)	C10—H10B	0.9600
C5—C8	1.459 (2)	C10—H10C	0.9600
C1—C2	1.385 (2)		
			
C8—O1—C9	117.56 (13)	H7B—C7—H7C	109.5
C1—N1—C1^i^	116.65 (16)	O1—C8—C5	110.47 (12)
N3—N2—C1	116.58 (11)	O2—C8—O1	123.32 (14)
C4—N2—N3	112.47 (11)	O2—C8—C5	126.21 (14)
C4—N2—C1	130.95 (11)	C1—C2—H2	121.6
C6—N3—N2	104.31 (11)	C3—C2—C1	116.88 (15)
N2—C4—C5	105.37 (11)	C3—C2—H2	121.6
N2—C4—C7	124.65 (12)	C2—C3—C2^i^	120.6 (2)
C5—C4—C7	129.91 (13)	C2—C3—H3	119.7
C4—C5—C6	105.49 (12)	C2^i^—C3—H3	119.7
C4—C5—C8	127.74 (13)	O1—C9—H9A	110.4
C6—C5—C8	126.76 (13)	O1—C9—H9B	110.4
N1—C1—N2	116.61 (12)	O1—C9—C10	106.82 (16)
N1—C1—C2	124.50 (13)	H9A—C9—H9B	108.6
C2—C1—N2	118.89 (12)	C10—C9—H9A	110.4
N3—C6—C5	112.36 (12)	C10—C9—H9B	110.4
N3—C6—H6	123.8	C9—C10—H10A	109.5
C5—C6—H6	123.8	C9—C10—H10B	109.5
C4—C7—H7A	109.5	C9—C10—H10C	109.5
C4—C7—H7B	109.5	H10A—C10—H10B	109.5
C4—C7—H7C	109.5	H10A—C10—H10C	109.5
H7A—C7—H7B	109.5	H10B—C10—H10C	109.5
H7A—C7—H7C	109.5		
			
N1—C1—C2—C3	−0.6 (2)	C1^i^—N1—C1—N2	−178.96 (14)
N2—N3—C6—C5	−0.20 (18)	C1^i^—N1—C1—C2	0.32 (12)
N2—C4—C5—C6	0.23 (15)	C1—N2—N3—C6	179.43 (12)
N2—C4—C5—C8	−178.84 (14)	C1—N2—C4—C5	−179.27 (13)
N2—C1—C2—C3	178.64 (11)	C1—N2—C4—C7	−2.0 (2)
N3—N2—C4—C5	−0.37 (16)	C1—C2—C3—C2^i^	0.29 (10)
N3—N2—C4—C7	176.90 (13)	C6—C5—C8—O1	6.9 (2)
N3—N2—C1—N1	149.52 (12)	C6—C5—C8—O2	−172.99 (17)
N3—N2—C1—C2	−29.80 (19)	C7—C4—C5—C6	−176.84 (15)
C4—N2—N3—C6	0.36 (17)	C7—C4—C5—C8	4.1 (3)
C4—N2—C1—N1	−31.6 (2)	C8—O1—C9—C10	176.62 (15)
C4—N2—C1—C2	149.05 (16)	C8—C5—C6—N3	179.07 (14)
C4—C5—C6—N3	−0.02 (18)	C9—O1—C8—O2	−1.4 (2)
C4—C5—C8—O1	−174.22 (14)	C9—O1—C8—C5	178.68 (13)
C4—C5—C8—O2	5.9 (3)		

Symmetry code: (i) −*x*+1, *y*, −*z*+3/2.

### Ethyl 1-{6-[4-(ethoxycarbonyl)-5-methylpyrazol-1-yl]\ pyridin-2-yl}-5-methylpyrazole-4-carboxylate Hydrogen-bond geometry (Å, º)

**Table:**

*D*—H···*A*	*D*—H	H···*A*	*D*···*A*	*D*—H···*A*
C7—H7*B*···O2	0.96	2.46 (1)	3.134 (2)	127 (1)
C7—H7*A*···O2	0.96	2.42 (1)	3.290 (2)	151 (1)
C6—H6···N3^ii^	0.93	2.63 (1)	3.388 (2)	140 (1)

Symmetry code: (ii) −*x*+1/2, −*y*+1/2, −*z*+1.
